# Architected materials for artificial reefs to increase storm energy dissipation

**DOI:** 10.1093/pnasnexus/pgae101

**Published:** 2024-03-26

**Authors:** Edvard Ronglan, Alfonso Parra Rubio, Alexis Oliveira da Silva, Dixia Fan, Jeffrey L Gair, Patritsia Maria Stathatou, Carolina Bastidas, Erik Strand, Jose del Aguila Ferrandis, Neil Gershenfeld, Michael S Triantafyllou

**Affiliations:** Department of Mechanical Engineering, Massachusetts Institute of Technology, Cambridge, MA 02139, USA; Center for Bits and Atoms, Massachusetts Institute of Technology, Cambridge, MA 02139, USA; ENSTA Paris, Institut Polytechnique de Paris, Palaiseau 91120, France; School of Engineering, Westlake University, Hangzhou 310024, China; Scinetics, Inc., Abingdon, MA 21009, USA; Center for Bits and Atoms, Massachusetts Institute of Technology, Cambridge, MA 02139, USA; Renewable Bioproducts Institute, Georgia Institute of Technology, Atlanta, GA 30332, USA; Sea Grant College Program, Massachusetts Institute of Technology, Cambridge, MA 02139, USA; Center for Bits and Atoms, Massachusetts Institute of Technology, Cambridge, MA 02139, USA; Department of Mechanical Engineering, Massachusetts Institute of Technology, Cambridge, MA 02139, USA; Center for Bits and Atoms, Massachusetts Institute of Technology, Cambridge, MA 02139, USA; Department of Mechanical Engineering, Massachusetts Institute of Technology, Cambridge, MA 02139, USA; Sea Grant College Program, Massachusetts Institute of Technology, Cambridge, MA 02139, USA

**Keywords:** architected reefs, wave energy dissipation, porous body hydrodynamics, drag amplification

## Abstract

Increasing extreme weather events require a corresponding increase in coastal protection. We show that architected materials, which have macroscopic properties that differ from those of their constituent components, can increase wave energy dissipation by more than an order of magnitude over both natural and existing artificial reefs, while providing a biocompatible environment. We present a search that optimized their design through proper hydrodynamic modeling and experimental testing, validated their performance, and characterized sustainable materials for their construction.

**Significance Statement**

Artificial reefs architected to provide substantially increased wave energy dissipation per unit projected area, provide a capability that does not exist today to protect coastlines against increasing storm intensity at reasonable cost. At the same time, the modular and porous design of the reefs provides shelter to marine life and makes installation easy.

## Introduction

Coastal erosion is a rapidly growing threat to both the natural environment and the 600 million people living in low-elevation coastal zones around the world (1). The loss of land and habitat, as well as the deterioration of infrastructure and economic assets, are just a few of the many challenges that follow (2–4). These challenges have been further intensified by climate change through rising sea levels, more frequent high-tide flooding, and powerful storm surges (1). As a result, without coastal protection or adaptation measures, a mean RCP8.5 scenario could result in an increase in the global land area, population, and assets at risk of flooding by 48, 52, and 46%, respectively by 2100 (1, 5, 6). The pressing need for effective coastal defense strategies that can protect our coastlines and communities has therefore never been more urgent.

## Background

### Extreme weather effects

Recent studies show that wave storms have intensified as a result of climate warming (7). For example, in 2023 severe storms battered California, damaging infrastructure and forcing people to flee away from the coast because the intensity of the storms has significantly increased since the 1970s. Using nearly a century of data, it was found that the occurrence of extreme significant wave height events during the 1996–2016 epoch is about twice that recorded between 1949 and 1969 (8). Combined with sea level rise, it is projected that by the end of the current century, even moderate wave storms will produce coastal impacts comparable to recent extreme winter wave events.

### Natural and artificial reefs

Coastal defense strategies to mitigate flooding and erosion have historically relied upon the implementation of hard engineering structures such as bulkheads, seawalls, breakwaters, tetrapods, gabions, and groins (9, 10). While these interventions can effectively reduce the impact of wave forces, they often come at a significant cost and can result in adverse wave reflection. Furthermore, such measures are constructed with little regard for the ecological requirements and functions of marine ecosystems, thereby impeding the natural replenishment of sediment and potentially exacerbating the risk of erosion and flooding in other regions (11).

Coral reefs in nature have been shown to attenuate up to 97% of wave energy serving as a unique natural coastal protection structure along nearly 71,000 km of coastline worldwide while also providing a myriad of other ecosystem services, including food and sustainable economies (12, 13). However, 60% of the world’s reefs are under immediate and direct threat from one or more local stressors, including rising ocean temperatures, overfishing, and coastal development. If no measures to reduce stressors are taken, 90% of coral reefs are expected to be in danger by 2030, and nearly all of them by 2050 (14). Other nature-based solutions, such as mangrove planting, offshore reefs, seagrasses, and sills, offer promising alternatives, but may not offer the necessary level of wave energy dissipation to address the pressing time constraints and required geographical scale for coastal protection (15–17).

In an effort to emulate the energy dissipation and marine habitat enhancement benefits of natural reefs, artificial reefs are being used. Artificial reefs typically consist of either sunken structures, such as oil and gas platforms, ships, and port structures, or they are specialized structures made of concrete, metal, plastic, tires, or rocks (18). As a result of their high complexity in terms of volume, area, and vertical relief, these structures have proven to support comparable levels of fish density, biomass, species richness, and diversity as natural reefs (19, 20). However, due to variability in planning and research, it was found that only 50% of artificial reef structures met their objectives in 2001 (21). Furthermore, even when constructed successfully, artificial reef structures often exhibit low wave energy dissipation per unit volume of material, necessitating the creation of large-scale structures to meet coastal protection requirements.

A crucial metric for artificial reefs is their ability to dissipate wave energy measured as dissipated energy nondimensionalized by the size of the reef. Since dissipation is caused by drag forces, an equivalent drag coefficient based on an equivalent projected area is the prime metric, which must be maximized. Nonstreamlined structures have relatively large drag coefficients, of the order of $C_{D}\approx 1$, when the drag coefficient, $C_{D}$, is referenced to the projected area in the direction of the flow. Hence, all currently used artificial reefs are expected to have a similar $C_{D}$ value, since they constitute such bluff structures.

In natural coral reefs, their wave energy attenuation capacity is based on having extensive areas of distributed coral at the reef flats with drag coefficients ranging from $C_{D}=0.02$ to 0.3 (22–24), combined with wave breaking at the reef crest (23).

### Architected reefs

Architected reefs are artificial reefs designed to dissipate wave energy at rates that are an order of magnitude higher than either natural or other artificial reefs. We target drag coefficients of the order of $C_{D}\approx 20$, which implies that they can achieve the same wave energy reduction as an artificial reef with $C_{D}\approx 1$, but with roughly 20 times less projected area. The comparison with natural reefs can be even more dramatic since reefs have a $C_{D}$ that is a fraction of 1, even when accounting for wave breaking.

**Fig. 1.**
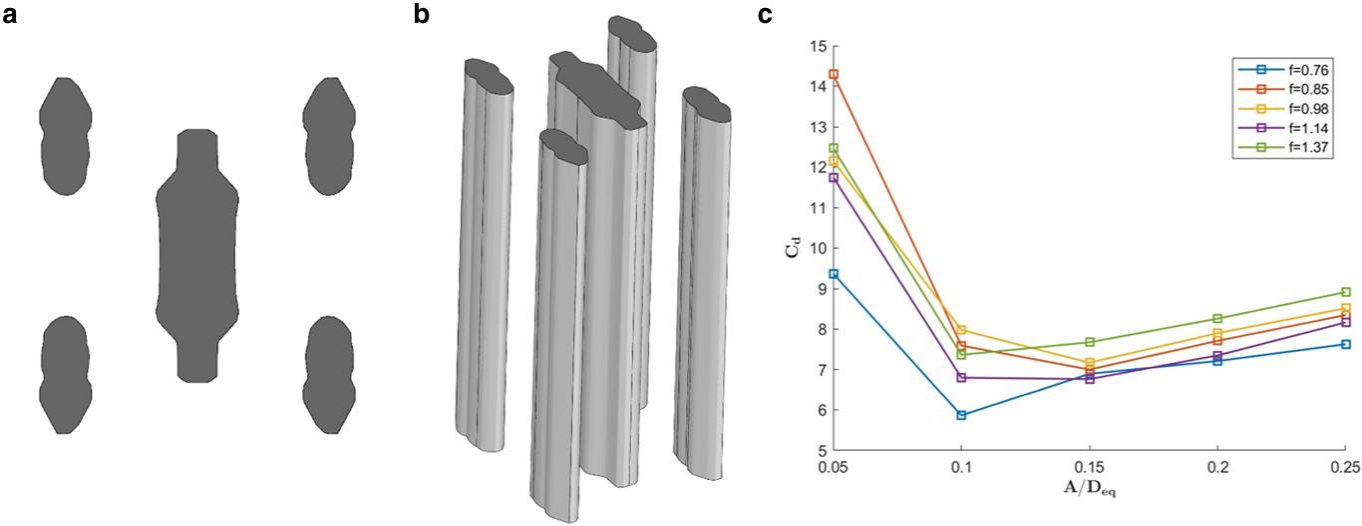
Illustration and experimental results for the baseline reef module: a) the cross-section, b) the configuration, and c) the experimental drag coefficient as function of the amplitude of oscillation, parametrically as function of the frequency.

To design an architected reef with such a dramatically increased rate of energy dissipation, we used the configuration shown in Fig. 1 as an initial model, consisting of five cylinders with a constant cross-section, monolithically extruded. This configuration is loosely based on a model studied in (25) that exhibited a large value of $C_{D}$ in oscillatory flow.

While this design is beneficial for large-scale vortex generation, it lacks the ability to adequately shelter marine life. Additionally, the manufacturing and underwater installation of such volumetric and solid structures would be highly complex and expensive. To address both issues, we introduced porosity to emulate the function of natural reefs, and hence studied their hydrodynamic properties. We proceeded to optimize the overall structure to further increase the drag coefficient relative to this baseline configuration.

Architected cellular materials effectively mitigate the challenges mentioned above due to their lightweight and high load-bearing capacity (26). They can also be discretely assembled (27), facilitating the construction of macroscopic structures using a building block strategy. Moreover, the inherent porosity of these macroscopic forms provides shelter for marine life. Considering these factors, we chose to design our ultimate artificial reef as a discretely assembled architected structure. This involved employing concrete unit cells to form the calculated optimized shape. The repeating unit cell, known as a voxel, was designed as a truncated pyramid. This particular geometry enables straightforward assembly along Cartesian directions while also facilitating a straightforward casting approach.

## Design of architected reefs

We developed optimized architected artificial reef designs for coastal protection against wave action, comprising multiple recurrent modules, through the following sequence of steps:

1. **Optimization:** We created a baseline reef module design, with a corresponding parametric Computer Aided Design (CAD) model, which was optimized utilizing combined computational fluid dynamics (CFD) simulations and experiments in the MIT Sea Grant towing tank, and Bayesian optimization.
2. **Porous building blocks:** We experimented with building the reef modules out of porous building blocks to make them easier to fabricate and more compatible with marine life by offering different spatial scales for settling, recruiting, growing, and sheltering. The resulting porous reef modules were also tested in the MIT Sea Grant towing tank and shown to have high-energy dissipation capacity.
3. **Experiments in waves:** We selected one of the best-performing modules and tested it in model scale in waves at the MIT towing tank confirming its extraordinary ability to dissipate wave energy.

### Optimization

We employed Bayesian optimization with Gaussian process regression as a surrogate model that selects the parameters to be tested at each iteration, a parametric model that generates a CAD model with the specified parameters, and a CFD program that evaluates the energy dissipation performance of the model and sends the result back to the optimization algorithm. This loop is repeated until the energy dissipation is maximized. CFD results are in parallel corroborated with experimental measurements.

#### Parametrization

We opted to design a reef made of a repeated pattern of simple-geometry modules. For the module, we chose the shape illustrated in Fig. 1 as a starting point, because it was previously found to have a high drag coefficient (25). We continued using this configuration throughout the optimization based on our understanding that high energy dissipation primarily occurs through a flow characterized by strong transient multidirectional jets. Such a flow can be achieved by pairing eddies from adjacent cylinders that make up the module; hence, a four-cylinder arrangement surrounding a central fifth cylinder is a relatively simple configuration that provides for four such pairing jets forming simultaneously at oblique angles in each half cycle. Also, the oval shape of individual cylinders, with the wide side facing the waves, is conducive to producing from the sharply curved edges of the cylinders large eddies that are nearly independent of Reynolds number and also insensitive to smaller scale perturbations, such as the voxels used for porosity, as well as marine growth effects. The four parameters we used allowed for an optimization leading to more effective production of pairing jets, more than doubling the drag coefficient of the original starting shape. We made the CAD model parametric and adaptable for optimization by leveraging the Solidworks API in Python.

#### Simulation

The CFD program used builds on the Boundary Data Immersion Method developed by Weymouth and Yue (28), which has been verified in subsequent studies (29, 30). This is an immersed boundary method that simulates the fluid–body interaction on a fixed Cartesian-grid. The program also uses implicit large-eddy simulation (iLES) to simulate turbulent flows, which is especially suited for modeling large-scale turbulent eddies, while not having to fit a body-fitted grid allows for efficient optimization.

To simplify the optimization effort, we studied harmonic waves of frequency ( ω=2πf) and amplitude (*A*) acting on an architected reef, and assessed the effect of amplitude and frequency of the waves. The dissipation of wave energy by the module is due to drag forces; hence, by utilizing the drag term of Morison’s equation, the energy dissipated over one time period for each module can be estimated as


(1) $$E_{d}=\int_{0}^{T}\int_{-H}^{q}\frac{1}{2}\rho C_{D}D_{\mathrm{eq}}u|u|u\;dz\;dt,$$

where *q* is the average distance from the water surface to the top of the reef module (if submerged, otherwise q=0), *H* is the water depth, u(z,t) is the horizontal component of the velocity at depth *z* and time *t*, *ρ* is the water density, $D_{\mathrm{eq}}$ is the equivalent diameter of a module, which is defined to be the diameter of a circular cylinder with the same area as the reef module (see Fig. S4 for illustration), T=2π/ω is the wave period, $C_{D}$ is the drag coefficient, and u(z,t) is the velocity of the wave at depth *z* and time *t*.

To further reduce the computational and experimental effort needed for optimization, we first studied the forces on a model of the reef module forced to oscillate at amplitude (*A*) and frequency (*ω*), rather than being subjected to waves. While there are differences of this model compared with the action of waves, which create a decaying amplitude of fluid oscillation as a function of depth, and involve both vertical and horizontal velocities, we show herein that the physical mechanisms are similar. Consequently, we employed CFD simulations in combination with experimental testing in the MIT Sea Grant 10-m towing tank, imposing horizontal harmonic oscillatory motions to a small-scale model of a reef module, to represent the effect of water waves:


(2) x(t)=Asin(ωt),

where *x* is the position of the reef module. We scaled the amplitude of motion by the ratio of the model length scale to the full-size length scale, and the frequency by the square root of the inverse of the same ratio, i.e. scaling the wavelength and using the dispersion relation. For example, for a wave of amplitude A=3m and frequency ω=0.72rad/s, or f=0.114Hz, we would target a reef module with equivalent diameter $D_{\mathrm{eq}}=6\;m$, so that $A/D_{\mathrm{eq}}\approx 0.5$. Hence, a reef model with equivalent diameter $D_{m}=7\;\mathrm{cm}$ would require a typical oscillation of A=3.5cm and a frequency of f=1.14Hz.

Since we optimize the energy dissipation per unit projected area, which is proportional to the drag coefficient, we calculate $C_{D}$ for harmonic oscillation and using Eq. 1 with constant *u* along *z* as


(3) $$C_{D}=\frac{\frac{1}{T}\int_{0}^{T}F(t)u(t)\;dt}{\frac{2}{3\pi }\rho D_{\mathrm{eq}}L(A\omega {)}^{3}},$$

where *L* is the length of the reef module.

The fixed parameters chosen for optimization were a frequency of f=0.76 Hz and amplitude $A/D_{\mathrm{eq}}=0.15$, leading to a Reynolds number of Re=3,510 when using the maximum oscillating velocity and equivalent diameter of the reef module. The reason for choosing a single frequency f=0.76 Hz was that the drag coefficient does not change substantially around this frequency value. Hence we chose the lowest relevant frequency to limit the resolution of the CFD simulations. An amplitude of $A/D_{\mathrm{eq}}=0.15$ was chosen as it was found to be high enough to generate fully developed flow. The drag coefficient at different amplitudes varies but is functionally related to *A*, as will be shown in this paper; so an optimization at $A/D_{\mathrm{eq}}=0.15$ optimizes the drag at higher/lower amplitudes as well.

### Porous building blocks

To validate the performance equivalence of a porous artificial reef (as described below) to a nonporous artificial reef, we conducted comparative experiments. As a first step before moving to voxel cells, we tested porous models made out of long circular steel rods to evaluate the effect of porosity without the influence of nonuniformity or sharp edges on flow. Using rods as building blocks also offers insights into the hydrodynamics of natural reefs made of cylindrical components. An optimized reef module populated with steel rods is depicted in Fig. S6.

Once we validated that porous models made out of steel rods perform hydrodynamically similarly to solid ones, we generated models of the voxel-based porous artificial reef as shown in Fig. S7. The models were fabricated using SLS Nylon printing, utilizing a Formlabs Fuse, which enabled the production of pieces with a resolution of 100μm. Given a certain optimized shape, we populated it with various sizes of truncated pyramids, with the associated parameters detailed in Fig. S8. The part name of the models to test will follow a nomenclature consisting of the parameters that Fig. S8 shows. A cell with *base square size* of 4 mm, with a *top square size* of 1.5 mm, a *cell-to-cell separation* of 0.8 mm, and a *floor thickness* of 0.5 mm will be named **B**40**T**15**C**08**F**05.

## Measurement

The experimental work was conducted in two different specialized towing tanks. The first tank located at MIT Sea Grant has an overall length of 10 m and was used to study the forces on the reef module forced to oscillate in quiescent water to verify the results of the CFD optimization. The second, a 30-m long towing tank, was used to study the full artificial reef in waves and its setup is described in the Supplementary material.

###  

#### Ten-meter towing tank for oscillating motion

The experiments involving sinusoidal motion were conducted at the MIT Sea Grant towing tank, which measures 10 m in length and 1 m in both width and height. The tank is equipped with a carriage system that can move in four degrees of freedom: two in the *x*-direction (steady forward, plus oscillatory forward), one in the *y*-direction, and one allowing for rotation about the *z*-axis. This setup enabled us to apply the sinusoidal motion described in Experimental results of the optimized reef section, and investigate different angles of attack for the reef modules. The parameters used for these experiments are listed in Table S1, where the amplitude and frequency are scaled as described in Experimental results of the optimized reef section. An illustration of the experimental setup is given in Fig. S1.

#### Thirty-meter towing tank for wave generation

The towing tank used for testing the full artificial reef in waves is 30 m long, 2.6 m wide, and 1.2 m deep. It features a wave generator that generates waves of controlled height and frequency, as well as a beach that absorbs the wave energy at the opposite end of the tank. The parametric values used for these experiments are given in Table S2 and illustrated in Fig. S4. These values were scaled based on actual storm conditions, CFD simulations, and results from the experiments conducted with the oscillating motion described in Experimental results of the optimized reef section. To create the artificial reef, we stacked reef modules in two rows, with the modules in each row placed in a staggered position to maximize obstruction to the flow induced by waves. To fit within the tank, this resulted in the first row having one more module than the second, as illustrated in Fig. S2.

The wave elevation before and after the reef was measured using eight probes, and their placements were based on previous studies (31–33). Specifically, four probes (P1, P2, P3, and P4) were positioned 5.9 m from the paddle and 6.6 m in front of the first row, while four probes (P5, P6, P7, and P8) were placed 5.4 m after the second row. At both locations, the probes were placed normal to the tank side as illustrated to the left in Fig. S3. To prevent interference from wave reflection, the wave elevation before the reef was measured until the waves reached the reef, and the wave elevation after the reef was measured until the waves reached the beach. An overview of the experimental setup is given in Fig. S3.

By measuring the wave amplitude before and after the artificial reef and using the expression [1] for the energy dissipation, we estimated the dissipation for the reef modules in waves, per module:


(4) $$E_{d}=\frac{4}{9}g\rho C_{D}D_{\mathrm{eq}}A^{3}.$$

For *N* rows, the percentage of wave energy reduced by the artificial reef as measured in the experiments, $d_{\%}$, is


(5) $$d_{\%}E_{f}TW_{\mathrm{tot}}=NE_{d},$$

where $E_{f}$ is the wave energy flux, *T* is the wave period, and $W_{\mathrm{tot}}$ is the width of each reef module including the distance between modules in a row ( $W_{\mathrm{tot}}=W+y_{s}$), as illustrated in Fig. S4. Solving for the equivalent drag coefficient, we obtain:


(6) $$C_{D}=d_{\%}\frac{18\pi }{16}\frac{gW_{\mathrm{tot}}}{N\omega^{2}D_{\mathrm{eq}}A}.$$

## Experimental results of the optimized reef

The optimization was conducted in two major stages, using two different baseline modules and sets of parameters, which is shown in Fig. S5 and Table S3. The model parameters included in the first round of optimization were the location (*x*- and *y*-coordinates) and the angle (*θ*) of the four outer bodies relative to the center body of the baseline module. The resulting experimental drag coefficient of the optimized reef with the corresponding cross-section and experimental model are presented in Fig. S5c. The drag coefficient is approximately twice that of the baseline module, a very substantial increase.

In a second stage, to further increase energy dissipation, we expanded the parametric space by including the lengths of the central and outer bodies as additional parameters. The experimentally measured drag coefficients of the best-performing configuration, given in Fig. S5f, show drag coefficients that are approximately twice those of the best-performing shape of the first optimization stage, a further dramatic increase in energy dissipation.

###  

#### Identifying the mechanism of large energy dissipation

**Fig. 2.**
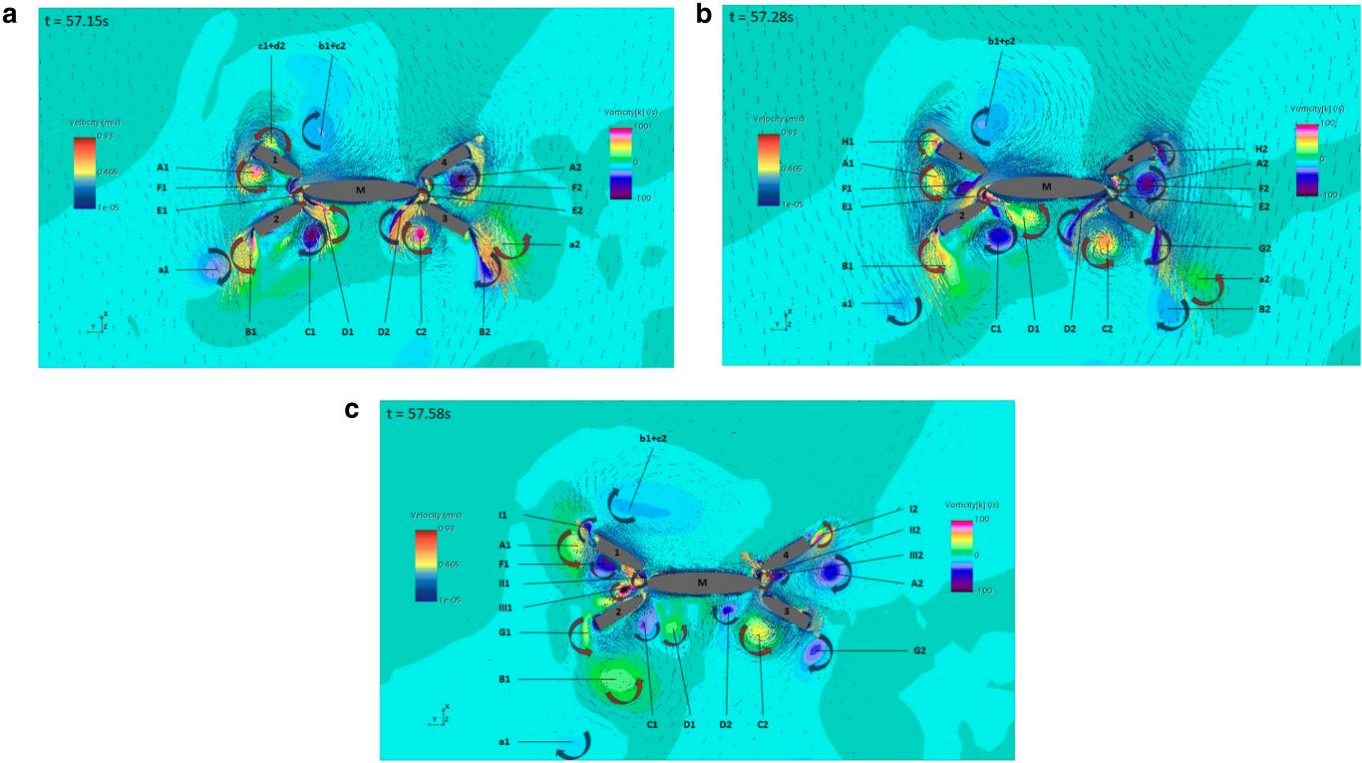
Visualization of velocity and vorticity in a horizontal cut of the flow at about mid-height of the module, illustrating the nearly symmetric flow around the best-performing reef module of the second optimization stage. Three snapshots are provided of the flow within the same cycle: in panel (a) at time *t* = 57.15 s, in panel (b) at *t* = 57.28 s, and in panel (c) at *t* = 57.58, to illustrate the vortex pairing processes and their evolution. The vorticity is illustrated with intensity coloring in the background, and the velocity is illustrated through flow vectors with associated intensity coloring. Clockwise and counterclockwise arrows are also added to highlight individual vortices. The naming of the vortices is done using lowercase letters for those formed in the previous half cycle, uppercase letters for those formed in the current half cycle, and Roman numerals for those formed in the subsequent half cycle. The numbers are odd for the left half and even for the right half of the flow. The simulations were done with f=0.76 Hz and $A/D_{\mathrm{eq}}=0.5$ in Simcenter Star CCM + 2022.1, resolving Detached Eddy Simulations with fully resolved boundary layers using SST-Menter k−ω turbulence model and a Convective Courant Number maintained below 1.

We clarify the mechanisms that lead to high drag coefficients by presenting flow visualizations for the best-performing module in the second optimization stage in Fig. 2. We show a horizontal visualization plane at about midheight of an oscillating module. For reference, we label the cross-section of the central body as M and those of the four outer bodies as 1, 2, 3, and 4, respectively. Vortices are denoted as C (clockwise) or CC (counterclockwise), and lowercase letters are used to represent vortices from the previous half cycle, uppercase letters denote forming vortices during the current half cycle, and Roman numbers are used for those of the next half cycle. Since the flow is nearly left–right symmetric, we will provide details only about the left half of the module.

In Fig. 2a at time t=57.15s, the flow moves from top to bottom, resulting in: a new vortex A1 (CC) from the left end of body 1; a new vortex B1 (CC) from the left end of body 2, accompanied by the vortex a1 (C) from the previous half cycle; a new pair of vortices between bodies 2 and M, specifically C1 (C) and D1 (CC); and another pair of vortices between bodies 1 and M, F1 (C) and E1 (CC). The pairs (C1, D1) and (F1, E1) induce intense jet flows, with a milder jet flow caused by the (a1, B1) pair. Figure 2b depicts the evolution at t=57.28 s, where the pairs (C1, D1) and (F1, E1) have grown and continue to be fed by the flow. By Fig. 2c at t=57.58 s, the flow reverses and starts to move from bottom to top. A new vortex pair (II1, III1) is forming between bodies 2 and M, nearing body 1, and a new vortex I1 emerges from the left end of body 1.

As shown by the visualizations in Fig. 2, very strong jets form through pairing of opposite-signed vortices. The jets have different directions, not aligned with the direction of body motion; also they persist for over half cycle resulting in a drastic increase in drag coefficient. Such observations are also consistent with previous research in shapes consisting of two cylinders (34, 35), showing that circular cylinders in side-by-side configuration induce higher drag coefficients due to the pairing of two oppositely signed vortices, formed from the sides of the two adjacent cylinders, which produces a jet flow. Similar findings were also established in the work with multiple cylinders used in the offshore industry (36).

We present herein results for the best-performing module design. Similar visualizations for the baseline design and for the first optimization stage modules, show significantly less jet formation, which results in lower drag coefficients.

### Testing porous building blocks

Porous, modular reef structures are highly desirable because they allow for easier deployment in the field, and provide shelter for marine life. Hence, we introduced porosity and modularity while ensuring that high dissipation is preserved. First, we introduced 2D porosity by constructing the bodies in each module from multiple, smaller diameter, parallel cylinders, placed at an adjustable distance from each other; and then we introduced 3D porosity.

In Table S4, we compare the experimental drag coefficient of porous modules made out of 3 mm steel rods placed at a distance equal to R/4 vs. that of nonporous modules at a frequency of f=0.98 Hz and an amplitude of $A/D_{\mathrm{eq}}=0.25$. Our results show that porous modules, both from the first and second optimization stages, experience a similar amplification of drag coefficient as nonporous modules, preserving the large-scale vortex formation as also noted in Nicolle and Eames (37). In fact, for the porosity tested, the porous modules consistently exhibit a higher drag coefficient than nonporous modules. The reason for this is the higher exposed area and the effect on the shedding angle in the detaching shear layers (37).

**Fig. 3.**
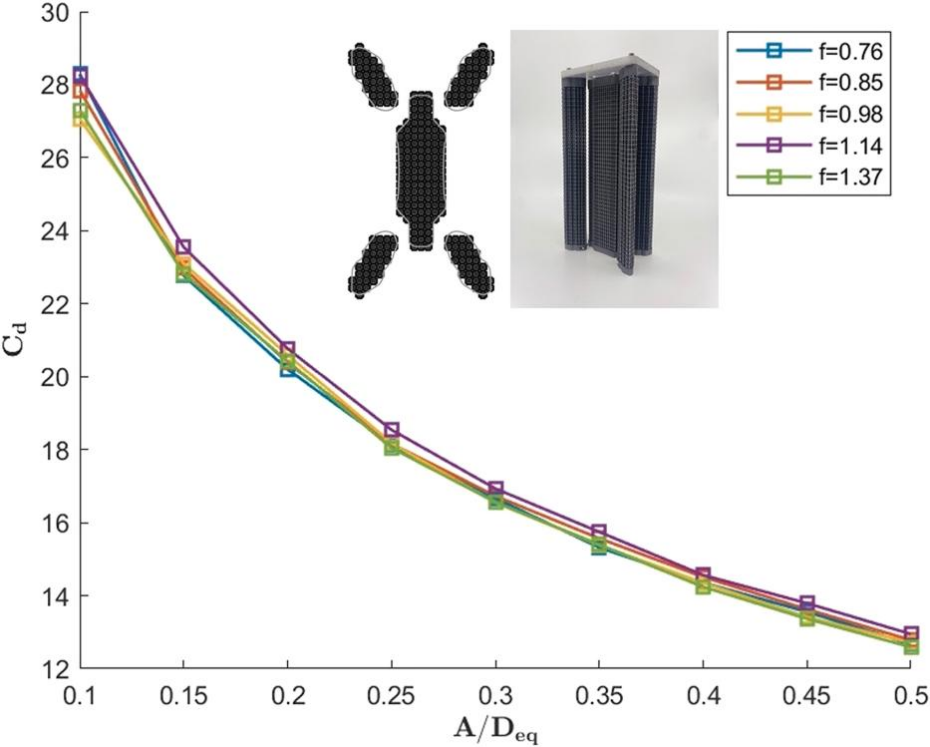
Experimental drag coefficient for reef module made out of voxels with part number **B**30**T**10**C**04**F**05, and with $x_{p}=0.6$, $y_{p}=1.3$, and $\theta_{p}=4/6$.

Three-dimensional porosity was introduced by utilizing optimized modular voxel building blocks. The part number of the voxels used was **B**30**T**10**C**04**F**05, and Fig. 3 provides the resulting drag coefficient together with an illustration of the experimental model. It is shown that the drag coefficient is close to that of the corresponding module made with rods, indicating that similar drag coefficients, and by extension similar flow structures result from both types of porosity.

The findings in this section are consistent with previous research, such as the work on unbounded flow around a localized circular array of multiple cylinders by Nicolle and Eames (37), where it was shown that, for low porosity, porous cylinders maintain a similar flow structure to solid cylinders that includes large-scale vortex formation; with a difference being that a wider shedding angle is found in the detaching shear layer.

### Artificial reef model tested in waves

To minimize cost, and given that similar forces were measured in porous modules, nonporous modules were utilized to build the artificial reef to be tested in waves. At the time of wave tank testing, the second optimization stage was not yet completed so we used the best-performing module in the first optimization stage. We compare the drag coefficient measured on reef modules in waves, with the experimental results of modules undergoing oscillatory motion.

**Fig. 4.**
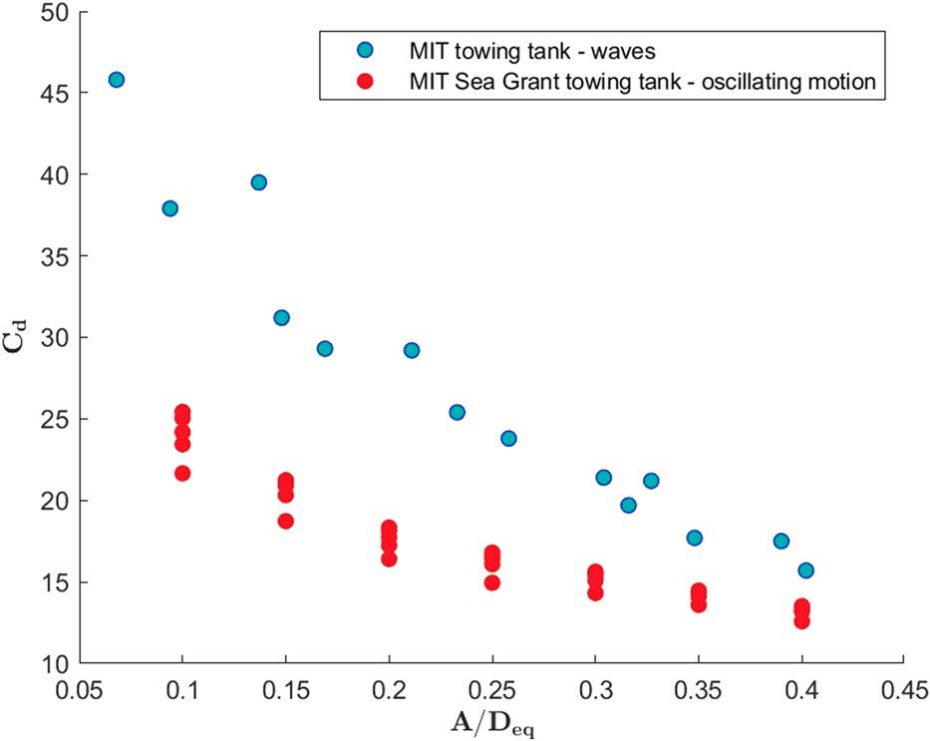
Drag coefficient of reef modules with $x_{p}=0.6$, $y_{p}=1.4$, and $\theta_{p}=3/6$ obtained in waves, compared to the drag coefficient obtained when the modules undergo a forced oscillatory motion.

As shown in Fig. 4, the drag coefficient in waves is conformed to be very high, reflecting significant energy dissipation. Also, compared with the drag measured in modules in forced motion, it is significantly higher. The reason for this is that in water waves the amplitude of fluid motion decays exponentially with water depth; hence, the amplitude of fluid motion to diameter ratio diminishes with depth, resulting in higher drag coefficients, which depend on 1/A, as explained in the Supplementary material. Since we employ the amplitude of motion at the water surface to derive an overall drag coefficient, the drag coefficient in waves is found consistently higher. The trends, in terms of amplitude and frequency dependence, remain the same.

## Discussion

The very high drag coefficients achieved through optimization herein are applicable to oscillatory flows. For a steady oncoming flow around a stationary structure, there is no mechanism for drag enhancement because no added mass-energy is available for conversion as explained in the Qualitative explanation of the high drag coefficients section in Supplementary material. In addition, we find high sensitivity of the process of vortex pairing to the timing of flow reversal; this implies that a steady flow superimposed on the oscillatory flow would degrade the drag amplification process. Hence, the results obtained here are applicable to wave storm energy attenuation, without simultaneous strong tidal surges.

Installing architected artificial reefs further offshore, before the wave breaking zone, provides a means for significantly reducing wave setup and overtopping, and hence sediment transport. As a result, we envision that these architected reefs are suitable for locations offering an appropriate bottom topography, viz. a sufficient depth combined with a mild slope near the shore such that the reef can be installed before wave breaking is initiated.

Natural reefs can protect coasts against intense wave storms and also provide shelter to abundant marine life. The nonporous architected reefs provide intense energy dissipation; while this is desirable for coastal protection, it is not conducive to sheltering marine life within the architected reefs due to the very strong unsteady flow patterns that form. To emulate the sheltering action of natural reefs, we introduced controlled porosity in the constituent building blocks, which was found to preserve their capability to dissipate wave energy, while providing protected spaces for marine life to shelter and grow. Indeed, the flow inside the porous modules is weak and characterized by the formation of small-scale vortices associated with wakes that are known to even have beneficial effects to the swimming effort of fish (38). The use of porosity architectures with fractal structure, as studied for example in Bai et al. (39), would provide a multiscale flow structure that is closer to that of natural reefs.

The sizing of an architected reef depends on the characteristics of the waves in a storm, and more specifically, to the significant wave height, providing the proper scaling parameters. The principal scaling parameter is the ratio of the characteristic wave amplitude to the equivalent diameter of the reef module. The significant wave amplitude (defined as the average one-third highest average amplitude for random waves) is the proper scaling parameter that allows to scale linearly the size of the reef module to a given storm. For the example above, when a storm has significant wave height 5 m, which is typical of sea state 6, see Ref. (40), we can target an amplitude to diameter ratio of 0.4, leading to the selection of reef modules with an equivalent diameter of 6.25 m. It is very helpful that frequency has a very weak effect on the reef dynamics, allowing a single parameter to dominate the scaling study and optimization of the reef in a set of storms. Although we have not conducted such an optimization in this study, multilayer reefs consisting of rows of modules with different equivalent diameters would be capable of strong energy dissipation for a range of storms with varying significant wave heights. This is clearly feasible due to the simple laws we established above.

In a preliminary assessment of the materials necessary to build the architected reefs, we considered five commercially available concrete materials that were found to offer (i) sufficient strength, (ii) low chloride permeability, and (iii) their biological and environmental impacts, in terms of leaching of lead, changes in seawater carbonate variables, and effects on survival of early life stages of marine species are minimal.

**Fig. 5.**
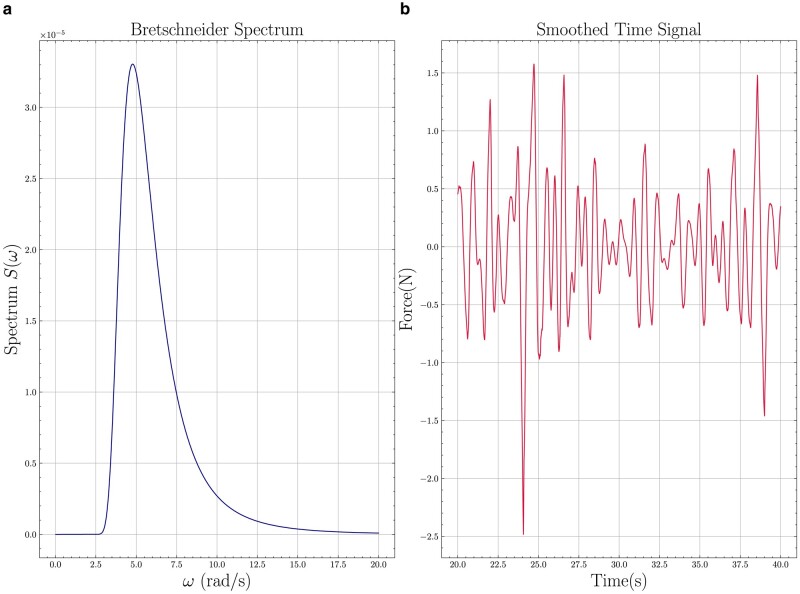
CFD results for a first generation reef module subject to random forced motion with a Bretschneider spectrum that is representative, in full scale, of sea state 5 (left panel). The calculated irregular force provides a drag coefficient of $C_{D}=18$. A 20-s segment of the overall force time trace is provided (right panel).

The artificial reefs will face random storm waves. To facilitate optimization, we conducted experiments and CFD in harmonic motion, which require far shorter testing times than a random input. The weak dependence on the frequency of harmonic waves and the fact that high drag coefficient is obtained for a wide range of amplitude to diameter ratios, allowed us to optimize the shape of the reef, utilizing effectively our computational and experimental resources. To show that the results are applicable to random seas, we run a CFD simulation of the first optimized reef model undergoing motion described by a Bretschneider spectrum, see Ref. (40), corresponding to sea state 6, with the following parameters: (i) significant wave height $H_{s}=5\;m$ and (ii) modal frequency $\omega_{m}=0.44\;\mathrm{rad}/s$, which at modal scale corresponds to significant wave height 4.2 cm and modal frequency 4.78 rad/s. Fig. 5 shows the shape of the spectrum used, a section of the force as function of time calculated with CFD, and the resulting drag coefficient is calculated as $C_{D}=18$. The significant amplitude to equivalent diameter ratio is $A_{s}/D_{\mathrm{eq}}=0.31$. The (roughly) equivalent value for the same reef model in harmonic motion at $A/D_{\mathrm{eq}}=0.31$ is around $C_{D}=16$, demonstrating that in random waves we obtain the same large values of the drag coefficient.

Freak waves constitute a hazard as their height often exceeds twice the significant wave height. For example, we referenced above (sea state 6) this would correspond to an amplitude of diameter ratio of 0.6. While the results in this paper show that the drag coefficients are still very high and will cause substantial energy dissipation, this poses additional design constraints that the structure must be capable of withstanding the wave forces at the highest storm to be expected. As for erosion due to waves, this occurs when repeated waves slowly remove sand and debris, combined by wave setup with breaking waves, while rarely occurring freak waves have a restricted effect, especially after significant attenuation by the artificial reef.

Biofouling is an issue for all structures in the ocean, especially when placed near the water surface. It is expected, and indeed desirable, to have marine life build within and around the architected reefs. The principal mechanism for increased drag is the formation of large-scale eddies, which have typical size comparable with the equivalent diameter of the constituent cylinders of the reef module, which is of the order of several meters (in our example above, the equivalent diameter is 6.25 m). This size is much larger than the size of marine growth, which has typical dimensions of the order of 0.2 m (41), making large eddy formation insensitive to typical marine growth, especially when the cylinders have an elliptical cross-section with high major to minor axes ratios, facing the flow with their high axis, such as the optimized shapes herein: The sharp curvature of the ends makes the vortex formation insensitive to surface perturbations , such as biofouling. Marine growth can decrease the porosity of the porous architected reef as marine life builds within the voxels of the modules. This does not degrade the wave energy reducing performance of the reefs, however, as shown for the monolithic reefs tested herein, which have zero porosity and yet very high drag coefficients.

## Supplementary Material

pgae101_Supplementary_Data

## Data Availability

All processed data will be preserved and be available on request.
