# Supplementary material for: Architected materials for artificial reefs to increase storm energy dissipation
Source: PNAS Nexus. 2024 Mar 26;3(3):pgae101. doi: 10.1093/pnasnexus/pgae101 (PMC10964131; doi:10.1093/pnasnexus/pgae101)
Supplement: pgae101_Supplementary_Data [file pgae101_supplementary_data.pdf]

PNAS Nexus, 2023, pp. 1–8

doi: DOI HERE
Advance Access Publication Date: Day Month Year
Paper

PAPER

Supplementary material for: Architected Materials for Artificial Reefs to Increase Storm Energy Dissipation

Edvard Ronglan,[a] Alfonso Parra Rubio,[b] Alexis Oliveira da Silva,[c] Dixia Fan,[d] Jeffrey L. Gair Jr.,[e] Patritsia Maria Stathatou,[b,g] Carolina Bastidas,[f] Erik Strand,[b] Jose del Aguila Ferrandis,[a] Neil Gershenfeld[b] and Michael S. Triantafyllou[a,f,*]

[a]Department of Mechanical Engineering, Massachusetts Institute of Technology, Cambridge, 02139, Massachusetts, USA, [b]Center for Bits and Atoms, Massachusetts Institute of Technology, Cambridge, 02139, Massachusetts, USA, [c]ENSTA Paris, Institut Polytechnique de Paris, Palaiseau, 91120, France, [d]School of Engineering, Westlake University, Hangzhou, 310024, Zhejiang, China, [e]Scinetics, Inc., Abingdon, 21009, Maryland, USA, [f]Sea Grant College Program, Massachusetts Institute of Technology, Cambridge, 02139, Massachusetts, USA and [g]Renewable Bioproducts Institute, Georgia Institute of Technology, Atlanta, 30332, Georgia, USA
*mistetri@mit.edu
FOR PUBLISHER ONLY Received on Date Month Year; accepted on Date Month Year

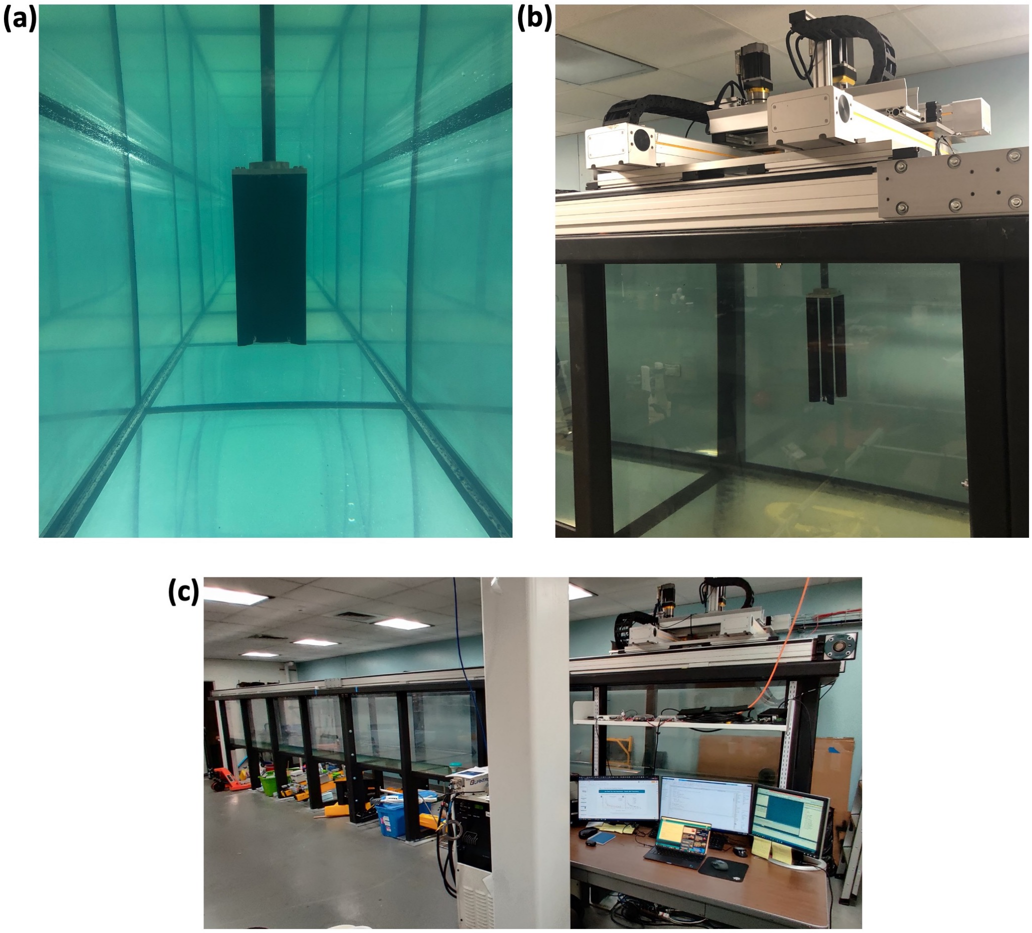

Fig. S1. MIT Sea Grant towing tank experimental set up. **(a)** includes a front view of reef module in the tank, **(b)** provides a close up of the automated carriage system, and **(c)** shows the overall tank.

**Parameter**	**Value**	**Description**
h	0.32 m	reef module height
D_{eq}	0.07 m	reef module equivalent diameter
H	0.9 m	water level in tank
$\frac{A}{D_{eq}}$	0.1-0.5	amplitude relative to equivalent diameter
f	0.76 Hz - 1.37 Hz	oscillation frequency

Table S1. Parameters for evaluating the drag coefficient of reef modules undergoing oscillatory motion.

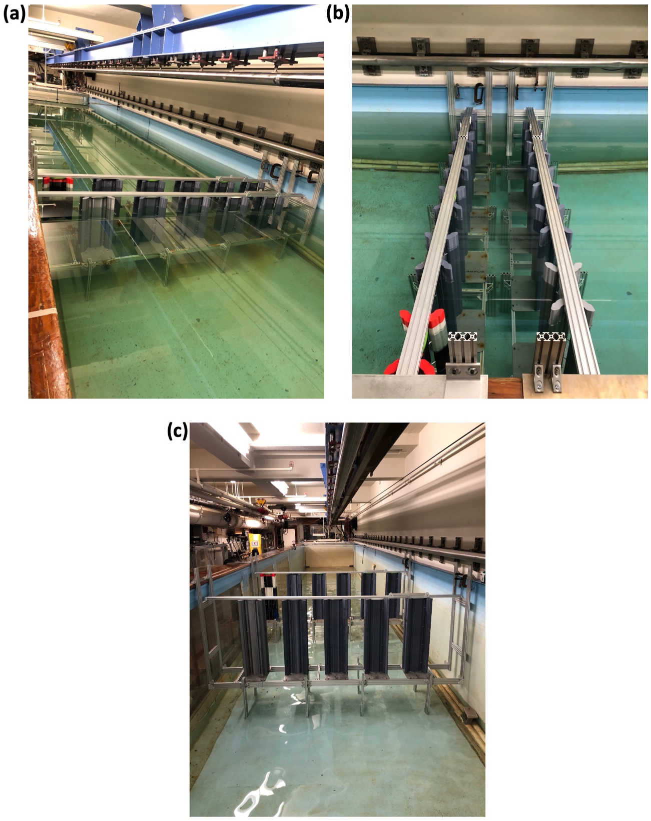

Fig. S2. Artificial reef in the 30-meter MIT towing tank for wave generation.

Parameter	Value	Description
H	1.15 m	water level in tank
A	0.01 m - 0.05 m	wave amplitude
f	0.8 Hz, 1 Hz and 1.2 Hz	wave frequency
h	0.78 m	reef module height
D_{eq}	0.12 m	equivalent diameter
W	0.24 m	width of reef module
y_s	0.18 m	spacing between units in a row
x_s	0.35 m	spacing between rows
N_y	6 and 5 (first and second row)	number of reef modules in a row
N_x	2	number of rows

Table S2. Parameters for testing in towing tank with waves.

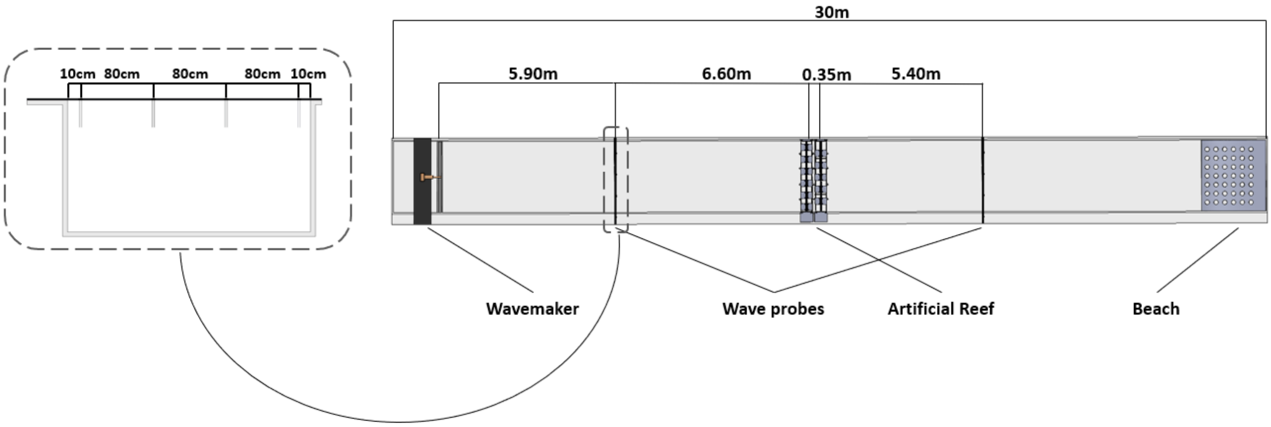

Fig. S3. Longitudinal section view of the towing tank for wave generation (right) and a cross-section view at the site of wave probes (left).

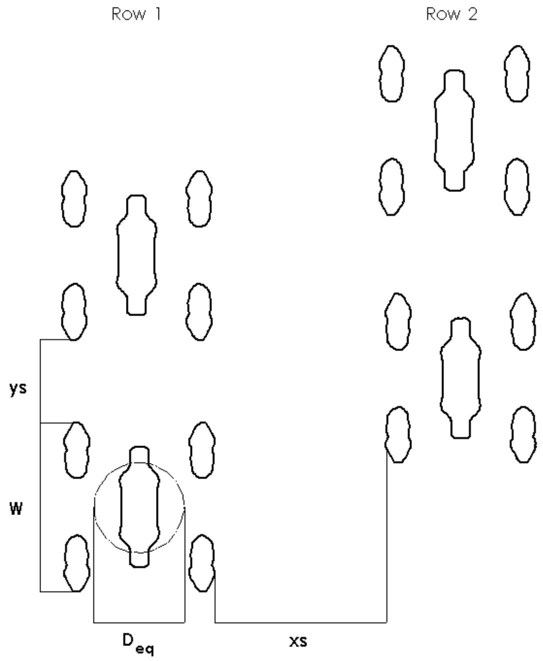

Fig. S4. Parameters for the artificial reef in the 30-meter MIT towing tank for wave generation.

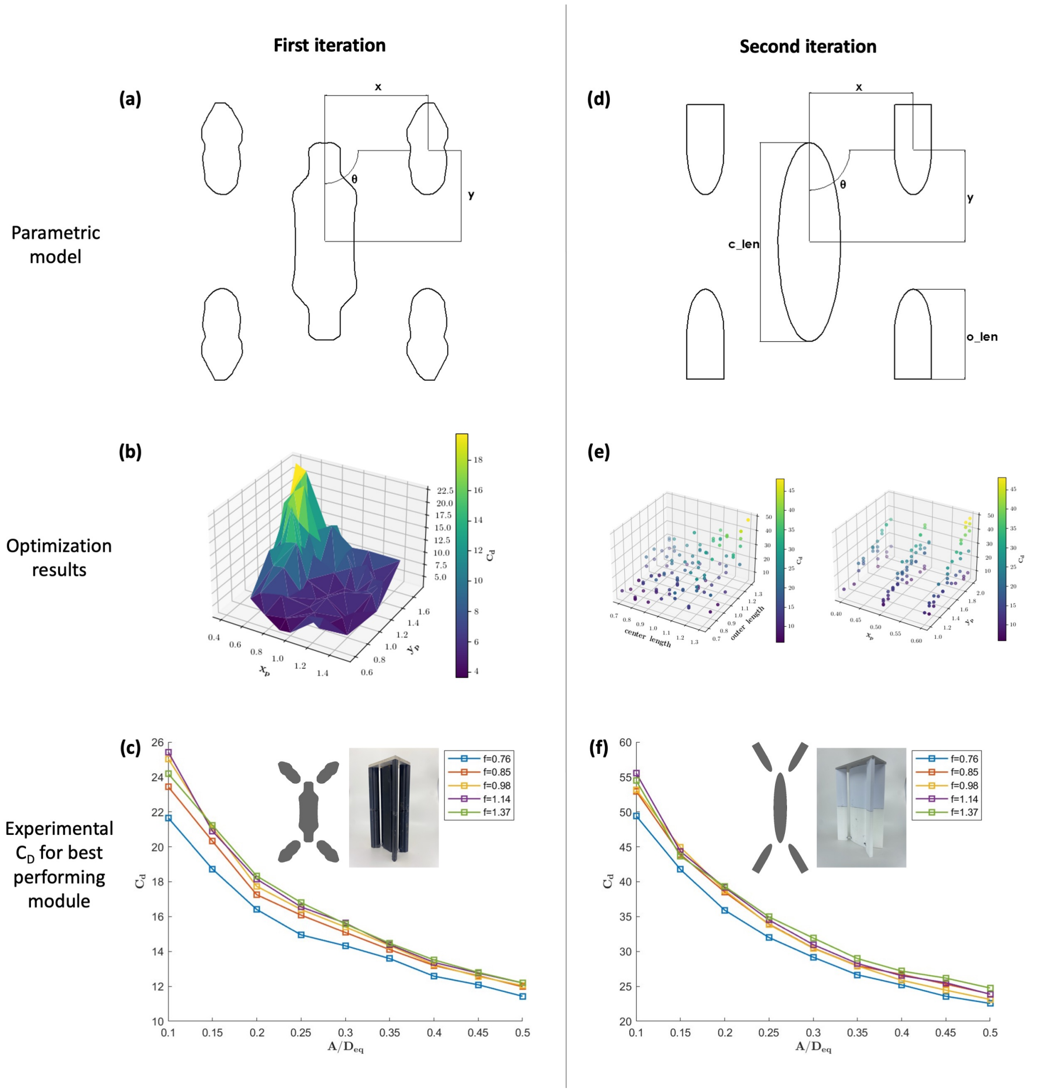

Fig. S5. Illustration of optimization parameters and results of two optimization stages. **(a)** shows the parameters for optimization, **(b)** includes the resulting drag coefficients plotted against two of the parameters (x_p and y_p), and **(c)** presents the experimental drag coefficient for the best-performing configuration for the first optimization stage. **(d,e,f)** includes the corresponding figures for the second optimization stage. The best performing modules are characterized by parameters $x_p = 0.6$, $y_p = 1.4$ and $\theta_p = 3/6$ for the first stage and $x_p = 0.6$, $y_p = 2.0$, $\theta_p = 4/6$, $c\_len = 1.3$ and $o\_len = 1.3$ for the second stage.

Parameter	Values First iteration	Values Second iteration
x_p	0.4, 0.5 , ... , 1.4, 1.5	0.4, 0.5, 0.6
y_p	0.6, 0.7 , ... , 1.6, 1.7	1.0, 1.1, ... , 1.9, 2.0
θ_p	1/6, 2/6, ... , 10/6, 11/6	3/6, 4/6, 5/6
c_len_p	-	0.7, 0.8, ... , 1.2, 1.3
o_len_p	-	0.7, 0.8, ... , 1.2, 1.3

Table S3. Parametric spaces for both optimization stages. The values of the parameters were given relative to their respective baseline reef module, with the following parametric values: $x = 43.7\ mm$, $y = 38.8\ mm$, $\theta = 90\ deg.$, c_len=83.66 mm and o_len=37.8 mm, where x, y and θ were common for both iterations and c_len and o_len were only used for the second stage.

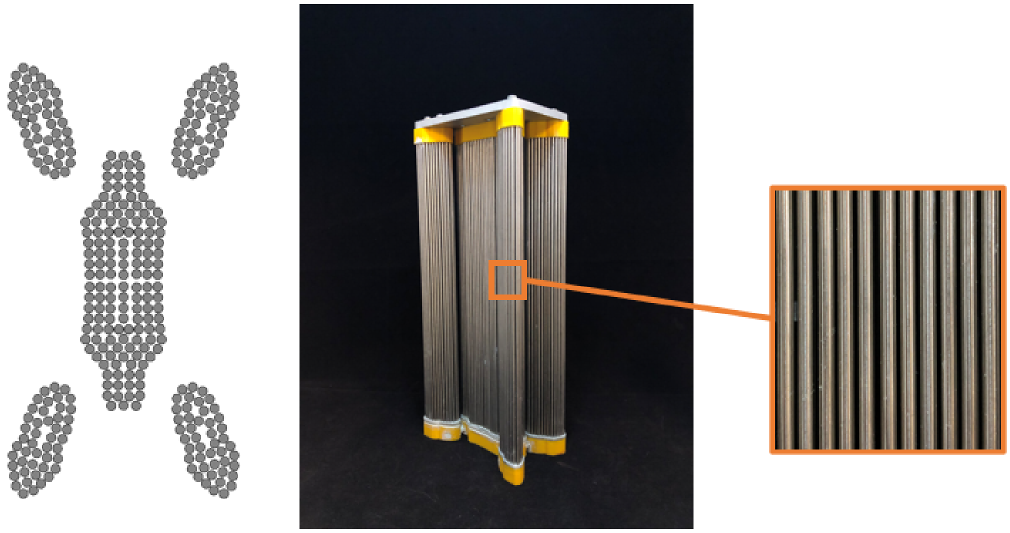

Fig. S6. Experimental model made out of 3mm equidistant steel rods, with R/4 distance, for a module with $x_p = 0.6$, $y_p = 1.3$, $\theta_p = 4/6$.

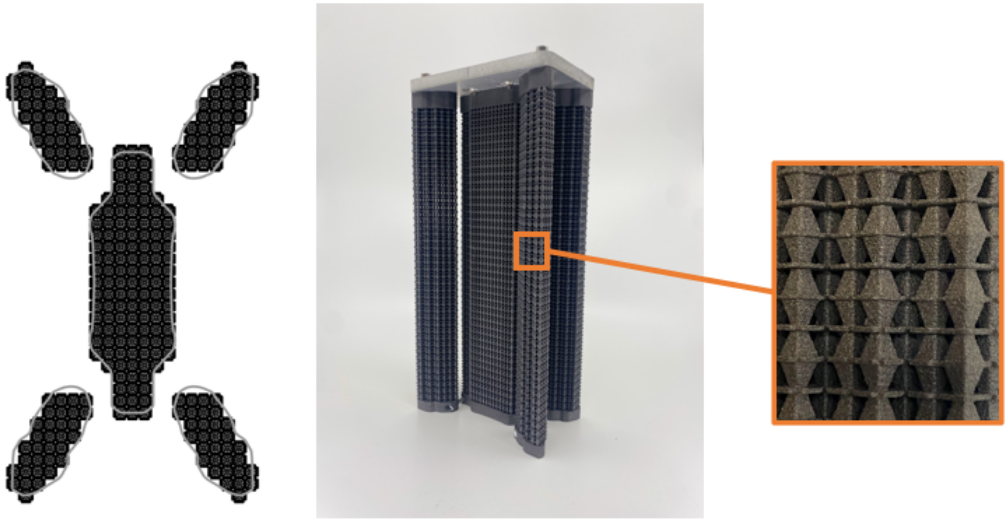

Fig. S7. Experimental model made out of voxels for a module with $x_p = 0.6$, $y_p = 1.3$, $\theta_p = 4/6$.

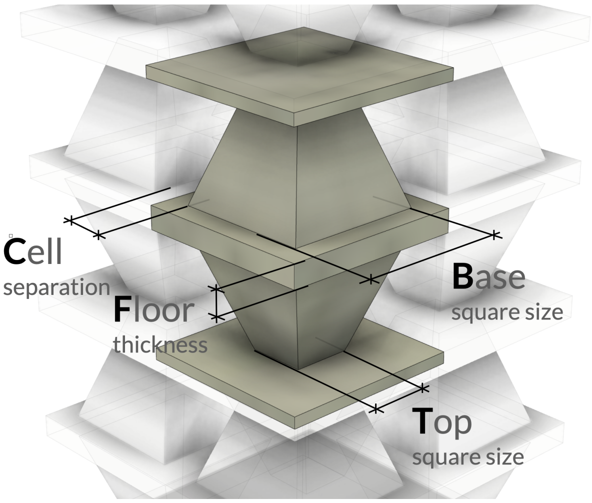

Fig. S8. Parameters for the truncated pyramid voxel building block.

Module	Drag coefficient porous	Drag coefficient non-porous
Baseline module	13.6	8.2
Optimized module	21.2	15.5

Table S4. Experimental drag coefficient at $A/D_{eq} = 0.25$ and $f = 0.98$ Hz for porous and non-porous modules. The two modules used are (a) the baseline module and (b) an optimized module with $x_p = 0.6$, $y_p = 1.3$ and $\theta_p = 4/6$. The porous modules were made out of steel rods with 3 mm diameter and $R/4$ clearance between adjacent rods. Fig. 1 illustrates the non-porous baseline module, while Fig. S1 presents the model used for the porous optimized module.

Qualitative Explanation of the high drag coefficients

The measured high drag coefficients herein, which are an order of magnitude higher than the typical drag coefficient in steady flow, require a qualitative explanation.

For a stationary bluff body within steady flow, a typical drag coefficient is of the order of $C_D \approx 1.0$, based on the projected area in the direction of the flow. The coefficient is a function of several parameters, such as the geometry of the body, the presence of sharp edges, and the Reynolds number. The specific values range, again typically, from about 0.2 for three-dimensional objects, to up to about 2.0 for two-dimensional objects with sharp edges. Conversely, for oscillating bodies, such as flexibly mounted bluff bodies placed within an oncoming cross-flow, the drag coefficient can be amplified by a factor of 5 or more, as the vibration of oscillation increases[2]. This amplification is a strong function of the reduced frequency $f^* = fU/D$, as well as the reduced amplitude $A^* = A/D$, where f is the frequency of oscillation, A the amplitude of oscillation, U the stream velocity, and D the projected diameter. Hence, it is possible to achieve higher drag coefficients in unsteady flows.

Moving to oscillatory oncoming flows, Williamson[7] found drag amplification when two cylinders of unequal diameter (the larger cylinder had a diameter equal to 3.5 times the smaller cylinder diameter) interact at small gap ratios. There is a marked increase of the in-line forces on both cylinders, up to a factor of 2 for the smallest gap ratio tested $g^* = 1$ (the gap ratio was based on the smaller cylinder diameter).

An insight into the source of drag or thrust increase due to unsteady motion can be found by exploring the mechanisms of conversion of added mass energy into the formation of vortical structures. In Weymouth & Triantafyllou[5, 6], it was shown that an accelerating body undergoing shape change can convert added mass-energy into energy-containing large eddies, with the boundary layer providing the requisite vorticity. These vortices can then induce significant thrust or drag. As outlined in Krueger & Gharib[3], moving vortices carry added mass energy; hence the conversion of the reef added mass energy as the flow accelerates, to that contained in the forming pairs of vortices, can provide an estimate of the structure and circulation of the eddies. Similar considerations apply to fish fast-starts, where it is shown that through body flexing the fish engages added mass energy that is then converted to form large vortices that provide axial forces to accelerate the fish rapidly[1, 4]. A different mechanism is employed by a fast-starting deflating octopus where added mass-energy is recaptured[6]. These cases involve conversion of added mass energy to either pressure gradients, or the formation of large-scale vortices, which can be manipulated to provide forces of desired direction and amplitude.

As shown in the main text, the pressure gradients induced by the oncoming waves cause the formation of multiple vortices, which can pair up to induce strong jets moving in various directions with respect to the oncoming waves, that can enhance wave energy dissipation.
To provide a simple qualitative explanation, we assume that the added mass-energy is converted entirely into energy contained in large-scale vortices, hence creating drag; we do not address the details of the mechanism through which this is achieved, as it has been discussed in the main paper through flow visualization, but we assume a total conversion. To further simplify the problem, we assume that the envelope of the reef shape can be approximated as a cylindrical body with an elliptical cross-section with minor semi-axis a and major semi-axis b, placed in an unsteady stream of velocity $u = u_o \sin(\omega t)$, where $u_o = A\omega$, A is the amplitude of oscillatory fluid motion, with the large semi-axis facing the flow. We define an equivalent diameter D_{eq} as that of a cylinder with the same area as the ellipse, viz. $D_{eq} = 2\sqrt(ab)$.

When the added mass energy is converted to form vortices, resulting in a drag force, we can write that over a cycle of period T:

$$m_a \int_0^T |\frac{du}{dt} u| dt = \frac{1}{2} \rho C_D D_{eq} \int_0^T u^2 |u| dt \tag{1}$$

where m_a is the added mass and C_D the drag coefficient. The absolute value within the integral is needed since the added mass energy is not recovered by the body. Taking $m_a \approx \pi b^2$, we find that:

$$C_D = \frac{3\pi}{8} \frac{b}{a} \frac{1}{(A/D_{eq})}. \tag{2}$$

Hence, we calculate, for example, that for $b/a = 5$, the drag coefficient for $A/D_{eq} = 0.25$ is $C_D = 24$, and for $A/D_{eq} = 0.5$ it is $C_D = 12$. Given that the elliptical shape is a substantial simplification of the reef shape, and the assumed ratio b/a is very approximate, these values are in good agreement with the values we find herein; also the trend of the drag coefficient depending on the inverse of the amplitude is fully explained.

References

1. M. Gazzola, W. M. Van Rees, and P. Koumoutsakos. C-start: optimal start of larval fish. *Journal of Fluid Mechanics*, 698:5–18, 2012.
2. A. Khalak and C. H. K. Williamson. Motions, forces and mode transitions in vortex-induced vibrations at low mass-damping. *J. Fluids Struct.*, 13:813–851, 1999.
3. Paul S. Krueger and M. Gharib. The significance of vortex ring formation to the impulse and thrust of a starting jet. *Physics of Fluids*, 15(5):1271–1281, 04 2003.
4. M.S. Triantafyllou, G.D. Weymouth, and J. Miao. Biomimetic survival hydrodynamics and flow sensing. *Annual Review of Fluid Mechanics*, 48:1–24, 2016.
5. G.D. Weymouth and M.S. Triantafyllou. Global vorticity shedding for a shrinking cylinder. *Journal of Fluid Mechanics*, 702:470–487, 2012.
6. G.D. Weymouth and M.S. Triantafyllou. Ultra-fast escape of a deformable jet-propelled body. *Journal of Fluid Mechanics*, 721:36–385, 2013.
7. C. H. K. Williamson. Sinusoidal flow relative to circular cylinders. *J. Fluid Mech.*, 155:141–174, 1985.
